# Patterns of population differentiation of candidate genes for cardiovascular disease

**DOI:** 10.1186/1471-2156-8-48

**Published:** 2007-07-12

**Authors:** Iftikhar J Kullo, Keyue Ding

**Affiliations:** 1 Division of Cardiovascular Diseases, Mayo Clinic, Rochester MN, USA

## Abstract

**Background:**

The basis for ethnic differences in cardiovascular disease (CVD) susceptibility is not fully understood. We investigated patterns of population differentiation (*F*_*ST*_) of a set of genes in etiologic pathways of CVD among 3 ethnic groups: Yoruba in Nigeria (YRI), Utah residents with European ancestry (CEU), and Han Chinese (CHB) + Japanese (JPT). We identified 37 pathways implicated in CVD based on the PANTHER classification and 416 genes in these pathways were further studied; these genes belonged to 6 biological processes (apoptosis, blood circulation and gas exchange, blood clotting, homeostasis, immune response, and lipoprotein metabolism). Genotype data were obtained from the HapMap database.

**Results:**

We calculated *F*_*ST *_for 15,559 common SNPs (minor allele frequency ≥ 0.10 in at least one population) in genes that co-segregated among the populations, as well as an average-weighted *F*_*ST *_for each gene. SNPs were classified as putatively functional (non-synonymous and untranslated regions) or non-functional (intronic and synonymous sites). Mean *F*_*ST *_values for common putatively functional variants were significantly higher than *F*_*ST *_values for nonfunctional variants. A significant variation in *F*_*ST *_was also seen based on biological processes; the processes of 'apoptosis' and 'lipoprotein metabolism' showed an excess of genes with high *F*_*ST*_. Thus, putative functional SNPs in genes in etiologic pathways for CVD show greater population differentiation than non-functional SNPs and a significant variance of *F*_*ST *_values was noted among pairwise population comparisons for different biological processes.

**Conclusion:**

These results suggest a possible basis for varying susceptibility to CVD among ethnic groups.

## Background

The human population is not homogeneous in terms of disease susceptibility and substantial differences in susceptibility to common chronic diseases such as cardiovascular disease (CVD), are present between self-identified ancestral/ethnic groups [1,2]. Significant differences in CVD prevalence were noted in the Seven Countries Study [3]. In the United States, African-Americans have a higher prevalence of hypertension [4] and hypertensive heart disease and significantly greater cardiovascular morbidity and mortality than Whites [5], whereas Japanese-Americans are less prone to CVD than Whites [6]. Differences in cardiovascular 'intermediate' phenotypes also occur among populations; for example, plasma lipid levels differ significantly between African-Americans and non-Hispanic whites, and plasma levels of C-reactive protein vary substantially between people of different ethnic origins [7]. The basis for ethnic differences in CVD susceptibility is not fully understood but it is likely that in addition to environmental factors, genetic factors contribute either by determining type or severity of risk factors, as well as the susceptibility to environmental/lifestyle risk factors [8-10].

Since different populations are subject to distinct environments, natural selection may produce population-specific allele frequencies. If a functional genetic variant exhibits significantly different pattern of geographic variation compared to a neutral variant, this may be indicative of different selective pressures across populations [11]. For instance, a given genetic variation may be adaptive under a local environmental stressor, which would increase the allele frequencies of this selected locus in a particular population and lead to a greater level of population differentiation [12,13]. A recent study suggested that differential susceptibility to hypertension may be due to differential exposure to selection pressures during the out-of-Africa expansion [14].

Natural selection alters the amount of differentiation between or among populations within a species so that a measure quantifying the differences in allele frequencies among human populations from diverse geographical regions – the *F*_*ST *_statistic – has been used to test for evidence of selection. *F*_*ST *_is a measure of the correlation between alleles in subpopulations relative to the alleles in the total population [15,16]. It is expected that local adaptation will lead to an increase in *F*_*ST*_, when comparing populations under different environmental pressures [17]. Multilocus scans in the human genome, using either single nucleotide polymorphisms (SNPs) [18] or microsatellite markers [19], that compare different populations for several loci, can identify genomic regions carrying a variant that results in a local adaptation [20]. Recently, Ryan *et al*. [21] investigated population differentiation among different functional classes of immunologically important genes and found significantly increased *F*_*ST *_in individual nonsynonymous SNPs of the intercellular adhesion molecule 1 (*ICAM1*) and Toll-like receptors (*TLR*) genes.

Population differentiation has particular relevance for studies of genetic susceptibility to complex diseases since many of the genes that are known to have been affected by natural selection are medically important [22]. Loci with an increased *F*_*ST *_should be considered high priority candidate genes for association studies of complex diseases as well as the study of local adaptation to environmental conditions. An example – provided by Rockman *et al*. [23] – is the increased frequency of high-expression allele (5T) of *MMP3 *due to positive selection in Europe but not elsewhere (i.e., a significant differentiation was noted between populations). This variant is associated with reduced arterial stiffness, resulting in lower CVD risk. Understanding genotypic difference among ethnic groups for these genes in relevant biological pathways will provide insights into ethnic differences in complex diseases that may be useful in the prevention and treatment of such diseases [1].

In the present study, using genotype data for three populations from HapMap [24] – Yoruban Africans (YRI), European Whites (CEU), and East Asians (CHB + JPT) – we investigated differences in the distribution of common variants (minor allele frequency ≥ 0.10) of 364 genes in etiologic pathways for CVD, and assessed patterns of population differentiation of these genes in the various biological processes underlying CVD. Our goal was to identify loci with high levels of population differentiation, as a step towards understanding the genetic basis of ethnic differences in cardiovascular risk.

## Results

### Genotype data for CVD candidate genes

Genotype data for 35,369 SNPs in 405 of 416 genes in etiologic pathways of CVD were available from HapMap; these included 24,391 SNPs in YRI, 22,751 SNPs in CEU, and 20,965 SNPs in CHB+JPT, respectively. Pairwise population comparisons of common variants (MAF ≥ 0.10) showed that a sizeable fraction (79% – 89%) of variants common in one population were present in another population. To avoid ascertainment bias, we focused on the co-segregating SNPs among three populations that had a minor allele frequency (MAF) ≥ 0.10 in at least one population and were in Hardy-Weinberg equilibrium, as suggested by Weir *et al*. [25]. In all, 15,559 such SNPs from 364 genes (87.5%) were identified and classified as putative functional SNPs [5' untranslated regions (5' UTR); coding (nonsynonymous) sites; and 3' untranslated regions (3' UTR)] and non-functional SNPs [coding (synonymous) sites; and intronic sites], based on NCBI SNP database [26] or UCSC genome browsers [27] (Table 1).

**Table 1:** The number of co-segregating SNPs for various genomic regions in three population samples from HapMap data set

Class	Number
5' flanking regions	1,224
Introns	13,601
Synonymous sites	217
Non-synonymous sites	140
3' flanking regions	377
Total	15,559

### Patterns of *F*_*ST *_of CVD candidate genes in three populations

#### Distribution of pairwise *F*_*ST *_values

**Figure 1:**
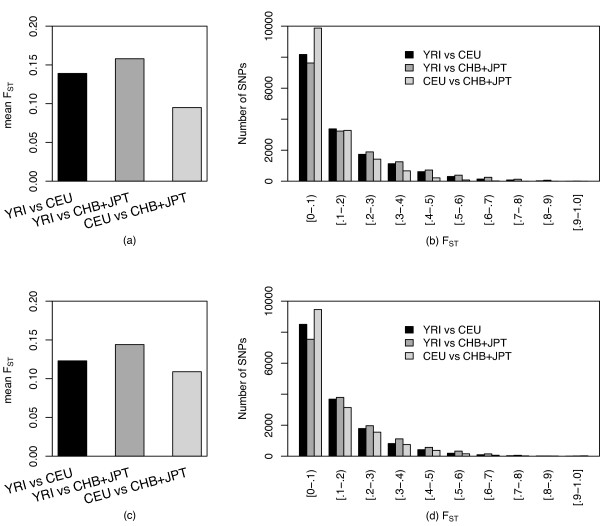
(a) Mean *F*_*ST *_in pairwise population comparisons in the observed data. (b) Distribution of *F*_*ST *_values in HapMap data set. (c) Mean *F*_*ST *_in pairwise population comparisons in the simulated data. (d) Distribution of *F*_*ST *_values in the simulated data set. YRI, Yoruba in Ibadan, Nigeria; CEU, Utah residents with ancestry from northern and western Europe, CHB, Han Chinese in Beijing, China, and JPT, Japanese in Tokyo, Japan

We first compared the distribution of *F*_*ST *_values of 15,559 SNPs among the three populations. The mean *F*_*ST *_for YRI vs. CEU was 0.139, for YRI vs. CHB + JPT, 0.158, and for CEU vs. CHB + JPT, 0.095 (Figure 1a). Thus, *F*_*ST *_between Africans and East Asians was slightly higher than the genome-wide average of *F*_*ST *_(0.10 ~0.15, i.e., the background *F*_*ST*_) previously noted to be present between sub-Saharan Africans, Northern Europeans, and East Asians [28-31]. The distribution of single-locus estimates of *F*_*ST *_values between two populations has an approximate χ^2 ^distribution (Figure 1b). The distribution of *F*_*ST *_between pairwise populations was significantly different (Kolmogorov-Smirnov test, *P *< 10^-16^). There was a higher proportion of low (< 0.10) pairwise *F*_*ST *_in CEU vs. CHB + JPT (63.5%), compared with YRI vs. CEU (52.5%) or CHB + JPT (49.0%). Larger *F*_*ST *_values (> 0.2) for common SNPs were observed in YRI vs. CEU (25.6%) and YRI vs. CHB + JPT (30.0%), but less often in CEU vs. CHB + JPT (15.4%).

We randomly selected 15,559 SNPs from the data generated by coalescent simulations (one MB region, 1,000 times, see Methods), which matched the characteristics of the observed data in terms of sample size, average *F*_*ST *_(Figure 1c), and MAF (i.e., ≥ 0.10 at least in one population). The simulated distribution of pairwise *F*_*ST *_(Figure 1d) was significantly different compared with the observed HapMap data (Kolmogorov-Smirnov test, *P *< 10^-10^). An excess of high-*F*_*ST *_values were present in the HapMap data for the set of genes in the present study, consistent with action of either genetic drift or natural selection and local adaptation leading to an increase in allele frequencies for the selected locus in a particular population [18]. It should be noted that the simulated *F*_*ST *_underestimated the *F*_*ST *_from the empirical data, consistent with the previous findings [32] and indicating that simulation did not perfectly predict *F*_*ST*_.

#### Distribution of pairwise *F*_*ST *_based on SNP functional classification

**Figure 2:**
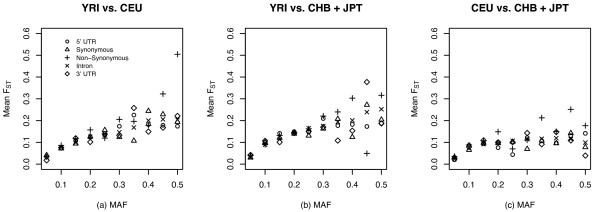
Mean *F*_*ST *_values for SNPs of different categories, conditioned on MAF. YRI, Yoruba in Ibadan, Nigeria; CEU, Utah residents with ancestry from northern and western Europe, CHB, Han Chinese in Beijing, China, and JPT, Japanese in Tokyo, Japan

To assess differences in the distribution of combined *F*_*ST *_values according to different categories of SNPs, we plotted the correlation between mean pairwise *F*_*ST *_and MAF according to the five different SNPs categories (Figure 2). The mean *F*_*ST *_values for SNPs of different categories conditioned on MAF are listed in Table S2 (see additional file 1). Using analysis of variance (ANOVA), we found that the pairwise mean *F*_*ST *_values in CEU vs. CHB + JPT varied significantly among different SNP categories (*P *= 0.019) by analysis of variance, but not in YRI vs. CEU (*P *= 0.273) and YRI vs. CHB + JPT (*P *= 0.124) (see Methods). In addition, pairwise mean *F*_*ST *_values between any two populations differed with MAF (*P *< 2.2 × 10^-16^), and there was a significant interaction of logarithm transformed MAF × category (*P *< 3.3 × 10^-5^), indicating that the effect of SNP category was modified by MAF.

Common, putative functional SNPs (i.e., SNPs in potentially functional genomic elements such as non-synonymous sites, 5' and 3' UTR) had systematically higher mean *F*_*ST *_values than SNPs in nonfunctional genomic elements (i.e., intronic and synonymous sites), although this was limited to SNPs with MAF ≥ 0.30. For example, when comparing YRI vs. CHB + JPT, mean *F*_*ST *_for common SNPs with MAF of 0.35–0.40 in non-synonymous sites (*F*_*ST *_= 0.303) was higher than that in synonymous (*F*_*ST *_= 0.124) and intronic sites (*F*_*ST *_= 0.201) (Figure 2b), although the effect was not statistically significant (pairwise comparison by 'multcomp' library in *R*) due to the limited number of non-synonymous sites. Much higher mean *F*_*ST *_values for non-synonymous SNPs with MAF of 0.45–0.50 were noted in YRI vs. CEU (*F*_*ST *_= 0.505).

#### Patterns of *F*_*ST *_in biological processes and functional pathways

**Figure 3:**
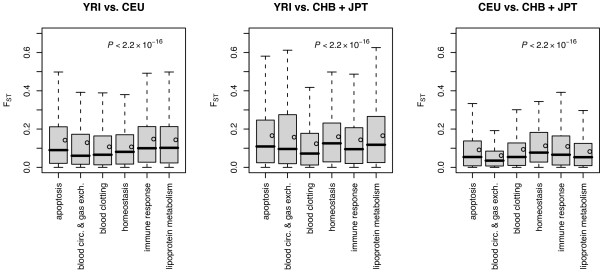
Boxplots of *F*_*ST *_according to biological processes. Points in the box are the mean *F*_*ST *_values. Outliers are not shown. *P *values were calculated by Kruskal-Wallis test. YRI, Yoruba in Ibadan, Nigeria; CEU, Utah residents with ancestry from northern and western Europe, CHB, Han Chinese in Beijing, China, and JPT, Japanese in Tokyo, Japan

We compared the distribution of *F*_*ST *_values for 364 genes in various biological processes (= 6) or functional pathways (= 37). The boxplots of *F*_*ST *_values in different biological processes are shown in Figure 3. Significant variation in *F*_*ST *_values was seen among different biological processes (*P *≤ 3.1 × 10^-15^, Kruskal-Wallis test).

In general, mean *F*_*ST *_for each biological process was significantly higher between Africans and non-Africans (especially between Africans and East Asians), in comparison with that between non-Africans (*P *< 0.05, pairwise *t *test) (Figure 3). The patterns of *F*_*ST *_suggested differential local factors operating on the selected biological processes among populations. For example, for genes in the 'blood circulation & gas exchange' pathway, the mean *F*_*ST *_value was 0.129 in YRI vs. CEU and 0.158 in YRI vs. CHB + JPT, but significantly lower (*F*_*ST *_= 0.062) in CEU vs. CHB + JPT. A similar pattern was also noted in the 'lipoprotein metabolism' genes (YRI vs. CEU: 0.144; YRI vs. CHB + JPT: 0.165; and CEU vs. CHB + JPT: 0.082).

Significant variation in *F*_*ST *_was also noted among the 37 functional pathways, (data not shown; *P *< 2.2 × 10^-16^, Kruskal-Wallis test). As expected, the mean *F*_*ST *_for each functional pathway was significantly higher between Africans and non-Africans, than between non-Africans. Most strikingly, genes in the 'Insulin/IGF-mitogen activated protein kinase kinase/MAP kinase cascade' pathway showed a relatively high *F*_*ST *_in all pairwise population comparisons (YRI vs. CEU: 0.194, YRI vs. CHB + JPT: 0.174 and CEU vs. CHB + JPT: 0.138). Also, a relatively high *F*_*ST *_between Africans and non-Africans was noted in the 'interleukin signaling pathway' genes (YRI vs. CEU: 0.192 and YRI vs. CHB + JPT: 0.210).

### Signatures of local adaptation

**Figure 4:**
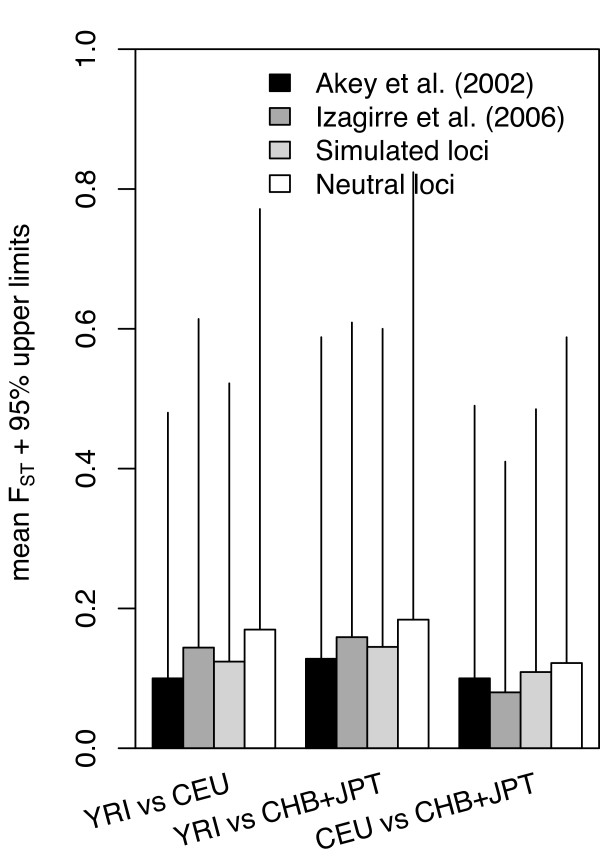
Mean and 95% upper limits of *F*_*ST *_distributions. Black, dark grey, and light grey bars represent the mean of *F*_*ST *_distribution found for the autosomal SNPs analyzed in Akey *et al*. [18], Izagirre *et al*. [47], and coalescent simulations, respectively. White bars represent the mean of *F*_*ST *_in the 'neutral' autosomal loci (14,792 SNPs) from the intergenic regions in this study. The 95% upper limits are placed on top of the mean value of *F*_*ST*_.

The HapMap data provides a genome-wide empirical distribution of *F*_*ST *_against which significance of *F*_*ST *_values can be evaluated, rather than based on theoretical computer simulations [33]. SNPs distant from genes are good candidates for neutral mutations since genes and their regulatory elements are more likely to be under selection than non-coding DNA [34]. We acquired the empirical 'neutral' distribution of *F*_*ST *_values from 289 intergenic regions across the autosomal genome (14,792 SNPs) and 17 intergenic regions across the X chromosome (372 SNPs) without considering the effect of MAF. For autosomal chromosomes, the 95% upper limits of *F*_*ST *_values were: YRI vs. CEU (= 0.602); YRI vs. CHB + JPT (= 0.640); and CEU vs. CHB + JPT (= 0.466) (Figure 4). The 95% upper limits of *F*_*ST *_values were higher for chromosome X – 0.729 (YRI vs. CEU), 0.828 (YRI vs. CHB + JPT), and 0.707 (CEU vs. CHB + JPT), respectively.

**Figure 5:**
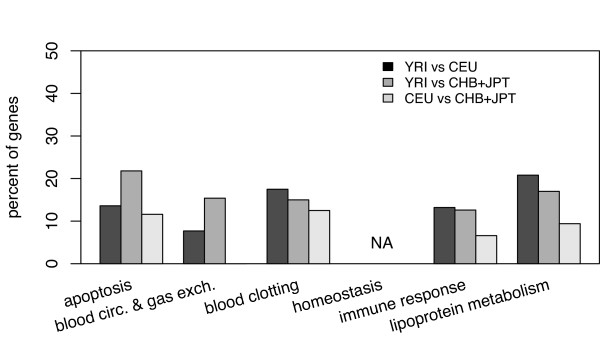
Percentage of genes in different biological processes with significantly high *F*_*ST *_(empirical *P *≤ 0.05) in at least one SNP.

We first calculated the significance level of *F*_*ST *_for each SNP locus. A small fraction of SNP loci showed a significantly higher *F*_*ST *_(*P *< 0.05) based on the empirical 'neutral' distribution of *F*_*ST *_values – 238 SNPs (1.53%) in YRI vs. CEU, 325 (2.09%) in YRI vs. CHB + JPT, and 164 (1.05%) in CEU vs. CHB + JPT, respectively. The number of genes with at least one unusually high *F*_*ST *_value according to biological process is shown in Table 2. The biological processes of 'apoptosis' and 'lipoprotein metabolism' showed an excess of genes (31.3% and 34.0%) with a significantly higher *F*_*ST *_(Table 2, Figure 5).

**Table 2:** Number of genes in different biological processes with significantly higher *F*_*ST *_(empirical *P *≤ 0.05) in at least one SNP

Biological process (gene number)	Pairwise population comparisons			**Total**
		
	YRI vs. CEU	YRI vs. CHB+JPT	CEU vs. CHB+JPT	
Apoptosis (147)	20 (13.6%)	32 (21.8%)	17 (11.6%)	**46 (31.3%)**
Blood circulation and gas exchange (13)	1 (7.7%)	2 (15.4%)	0 (0%)	**2 (15.4%)**
Blood clotting (40)	7 (17.5%)	6 (15.0%)	5 (12.5%)	**9 (22.5%)**
Homeostasis (9)	-	-	-	**-**
Immune response (151)	20 (13.2%)	19 (12.6%)	10 (6.6%)	**35 (23.2%)**
Lipoprotein metabolism (53)	11 (20.8%)	9 (17.0%)	5 (9.4%)	**18 (34.0%)**
**Total (364)**	**51 (14.0%)**	**63 (17.3%)**	**32 (8.8%)**	**110 (30.2%)**

**Figure 6:**
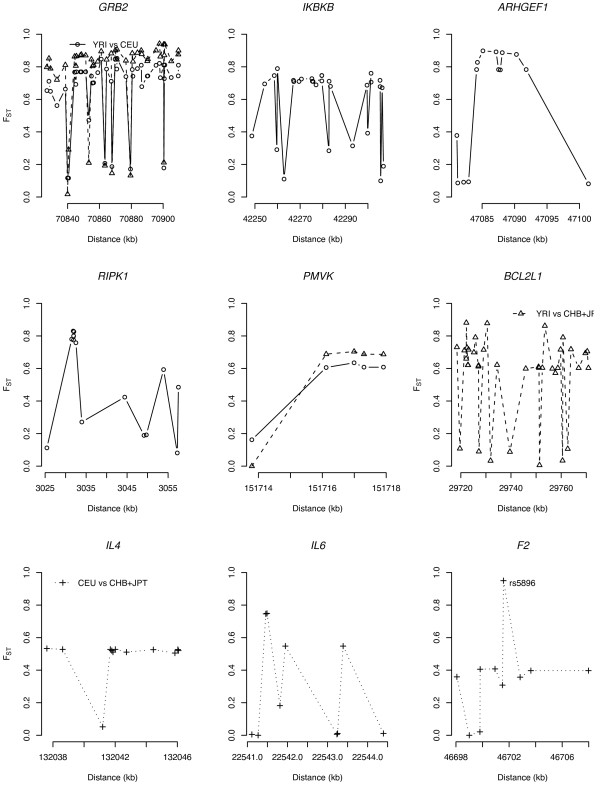
*F*_*ST *_profile for nine genes with a significantly higher weighted-average *F*_*ST*_. The X-axis indicates the chromosomal position (kb). See Table 3 for gene names. The average recombination rate (cM/MB) for the genes is: *GRB2*, 1.78; *IKBKB*, 0.76; *ARHGEF1*, 0.81; *RIPK1*, 2.06; *PMVK*, 1.07; *BCL2L1*, 0.86; *IL4*, 0.94; IL6, 1.10; *F2*, 0.66.

We also calculated a weighted-average *F*_*ST*_, combining information over loci [35] that summarizes the levels of interpopulation differentiation in each gene. Genes with a significantly higher weighted-average *F*_*ST *_are shown in Table 3. In total, there were signatures of local adaptation in nine genes (2.5%) – four genes in YRI vs. CEU and three genes in YRI vs. CHB + JPT, and three genes in CEU vs. CHB + JPT. Most of the genes are involved in 'immune response' (*GRB2*, *IKBKB*, *IL4*, *IL6*) and 'apoptosis' (*ARHGEF1*, *RIPK1*, *BCL2L1*, *IL4*, *IL6*), as well as one gene each in 'blood clotting' (*F2*) and 'lipoprotein metabolism' (*PMVK*). The distribution of *F*_*ST *_along the sequence for these genes is shown in Figure 6, indicating multiple SNP loci with a significantly high *F*_*ST*_.

**Table 3:** Genes with a significantly high weighted-average *F*_*ST *_(*P *≤ 0.05)

Symbol	Gene name	YRI vs. CEU		YRI vs. CHB + JPT		CEU vs. CHB + JPT		Biological processes
					
		*F*_*ST*_	*P*	*F*_*ST*_	*P*	*F*_*ST*_	*P*	
*GRB2*	growth factor receptor-bound protein 2	0.720	0.035*	0.828	0.023*	0.048	0.556	Immune response
*IKBKB*	inhibitor of kappa light polypeptide gene enhancer in B-cells, kinase beta	0.658	0.043*	0.555	0.071	0.034	0.605	Immune response
*IL4*	interleukin 4	0.262	0.220	0.205	0.329	0.512	0.043*	Immune response/Apoptosis
*IL6*	interleukin 6	0.108	0.478	0.431	0.119	0.570	0.037*	Immune response/Apoptosis
*ARHGEF1*	Rho guanine nucleotide exchange factor 1	0.769	0.030*	0.532	0.078	0.149	0.263	Apoptosis
*RIPK1*	receptor (*TNFRSF*)-interacting serine-threonine kinase 1	0.642	0.045*	0.544	0.074	0.070	0.456	Apoptosis
*BCL2L1*	BCL2-like 1	0.278	0.204	0.679	0.043*	0.265	0.127	Apoptosis
*PMVK*	phosphomevalonate kinase	0.563	0.059*	0.652	0.048*	0.097	0.380	Lipoprotein metabolism
*F2*	coagulation factor II	0.413	0.109	0.536	0.077	0.473	0.049*	Blood clotting

* Genes with a significantly higher weighted-average *F*_*ST*_

## Discussion

The most direct way to study whether genetic risk factors vary among ethnic groups is to determine whether disease susceptibility variants differ in frequency and/or effect among groups [36]. Several studies have demonstrated that the genotype frequencies of SNPs in candidate genes for cardiovascular diseases (CVD) differ among populations [2,9,37]. Lanfear *et al*. [9] found higher frequencies of disease-associated genotypes in African-Americans than in European-Americans for polymorphisms in *GJA4*, *SERPINE1 *(*PAI*-*1*) and *MMP3*. Two nonsense mutations in *PCSK9 *that lead to lower plasma levels of low-density lipoprotein cholesterol are relatively common in African-Americans (2%) but rare in European-Americans (< 0.1%) [37]. Significant differences in allele frequencies were noted in the polymorphisms of *IL2*, *IL6*, and *IL10 *among Blacks, Whites, and Asians [38]. In a meta-analysis, Ioannidis *et al*. [2] assessed 43 validated gene-disease associations across 697 study populations of various ethnicities and found that frequencies of polymorphisms in seven cardiovascular disease genes – *APOE*, *ACE*, *ITGB3*, *MTHFR*, *F2*, *PON1*, and *PON2 *– varied significantly between ethnicities (I^2 ^≥ 75%; I^2 ^being a measure of the extent to which the heterogeneity is not due to chance) [2]. These loci (except *APOE*) showed a large heterogeneity of 'race'-specific frequency of polymorphisms. In addition, a disease-associated mutation may be present at high frequency in one population but virtually absent in another, an example being a variant in the *SCN5A *gene (associated with cardiac arrhythmia) [39], which is present in African-Americans at an allele frequency of 0.132 and is not found in Europeans and Asians. However, the *F*_*ST *_value may not be a 'sensitive' test and genes implicated in CVD susceptibility may not lead to exceptionally elevated *F*_*ST *_values. For example, the *F*_*ST *_value was not significantly higher for the nonsense mutation in *PCSK9 *when comparing African-Americans (allele frequency: ~2%) and European-Americans (frequency < 0.1%).

Gene variants that interact with geographic- or population-specific environmental factors may be under strong positive or diversifying selection pressure [40]. A measure of population differentiation (i.e., *F*_*ST*_) has been used to quantify the degree to which populations are subdivided for particular genetic variants. While other measures, such as the nearest-neighbor statistic (*S*_*nn*_) [41], and *c *parameter (i.e., measuring how isolated a population has been) [42] are used to identify the level of population differentiation, the unbiased estimator of *F*_*ST *_is simple and easy to calculate. We calculated *F*_*ST *_for SNPs in a set of genes in causal pathways for CVD, to identify the patterns of differences for allele frequencies from one ethnic group to another.

To avoid false positive results due to genotyping error and ascertainment bias [18,25,43], we studied SNPs that were in Hardy-Weinberg equilibrium, had a minor allele frequency (MAF) of ≥ 0.10 in at least one population (i.e., common SNPs), and cosegregated in all three populations [25]. The relatively low genotype error rate (0.3%) in HapMap data [33] has likely had a limited impact on estimates of *F*_*ST *_[18]. Consistent with previous reports [44], most common SNPs were shared between populations in the present study and 10–20% of the common variants present in one population are not necessarily common in the other population (i.e., private SNPs), especially when comparing Africans and non-Africans. However, whether the 'private' or shared common SNPs contribute to ethnic differences of CVD risk needs further investigation. The approximate chi-square distribution of *F*_*ST *_(Figure 1b) is similar to the distribution for the entire HapMap data [25]. The distribution of pairwise *F*_*ST *_is similar for the YRI vs. CEU and YRI vs. CHB + JPT, consistent with previous reports [28,33,44]. The lower level of population differentiation in CEU vs. CHB + JPT supports a recent split between these two populations.

Given that candidate loci with large *F*_*ST *_values might have undergone local adaptation [18,45], we hypothesized that *F*_*ST *_values would be higher in putatively functional variants than in putatively nonfunctional variants. Common functional SNPs with large *F*_*ST *_values may influence variation in disease susceptibility among different populations. A high divergence of allele frequency was noted among putatively functional (e.g., nonsynonymous sites and SNPs in 5', and 3' UTRs) and non-functional SNPs (Figure 2). In addition, the pattern varied in different pairwise population comparisons and was most obvious for the Africans vs. non-Africans comparison. An extreme example is a non-synonymous SNP in *F2 *(rs5896, Met165 → Thr) with higher *F*_*ST *_in YRI vs. CHB + JPT (*F*_*ST *_= 1.000) and CEU vs. CHB + JPT (*F*_*ST *_= 0.950), at a MAF of 0.00, 0.05, and 1.00 in YRI, CEU and CHB + JPT, respectively. This finding has also been reported in a prior analysis of the Phase I HapMap data [33].

To provide a reasonable biological explanation for the *F*_*ST *_values, we considered the sampling distribution of the *F*_*ST *_estimates. One of the methods is based on numerical sampling or permutation procedures so that *F*_*ST *_is estimated and the proportion of values larger than or equal to the one estimated from the observed data set will yield the unbiased *P*-value of the test [46]. Yet another method involves the use of variances in actual values of *F*_*ST *_to detect regions of exceptional *F*_*ST *_values, defined as population-average values more than three standard deviations from the chromosomal average [25]. An alternative strategy is based on coalescent theory that simulates the histories of the samples or the populations. An expected distribution of *F*_*ST *_for 15,559 simulated SNPs was generated under the calibrated demographic model [32] (Figure 1c~d). Recently, an empirical distribution has been used to test the significance level of *F*_*ST *_[18,47,48]. Instead of whole-genome empirical distribution of *F*_*ST *_[18,34], we used an empirical distribution of *F*_*ST *_from 'neutral' loci in 289 and 17 intergenic regions from autosomal chromosomes and X chromosome, respectively. The number of SNPs for empirical neutral distribution (14,792 from autosomal chromosomes and 372 from the X chromosome) takes into account the multiple testing incurred in our evaluation of 15,559 SNPs. This 'neutral' empirical distribution of *F*_*ST *_is most likely shaped by only demography and therefore the *P *values of *F*_*ST *_estimated from the empirical distribution may represent a more reliable indicator of selection. The mean and 95% upper limits of this 'neutral' distribution are slightly higher than the previously used 'neutral' empirical [18,47] or the simulated distribution (Figure 4), indicating the statistical test using the empirical distribution is more conservative. Although none of the SNPs or the genes remained significant after correction for multiple testing using false discovery rate [49], the method of population differentiation can used as an exploratory tool for detecting local adaptation [47].

Genes are subjected to different evolutionary constraints depending on their biological functions and genes with a higher population differentiation are likely to have been more readily influenced by the environment [40]. For instance, Grossman *et al*. [50] found that *F*_*ST *_values for 'apoptosis' genes among Ashkenazi, Sephardic and Arab Israelis are low. In the present study, most striking was the high mean *F*_*ST *_in the biological process of 'apoptosis' (*F*_*ST *_= 0.166) and 'lipoprotein metabolism' (*F*_*ST *_= 0.165) between YRI vs. CHB + JPT, but lower *F*_*ST *_in CEU vs. CHB + JPT (0.091 and 0.082, respectively) (Figure 3). The patterns suggested that differential environments pressures may have accounted for the varying *F*_*ST *_among biological processes for different populations.

The biological processes of 'apoptosis' and 'lipoprotein metabolism' showed an excess of high *F*_*ST *_values, which may be the result of local adaptation or genetic drift (Table 2, Figure 5). The combined information of *F*_*ST *_over loci provides a means to quantify the level of population differentiation in a given gene (Figure 6) [35]. A summary of functions for these genes relevant to CVD is shown in Table S3 (see additional file 1). For three gene loci – *F2, IL4*, and *IL6 *– significantly higher *F*_*ST *_values in CEU vs. CHB + JPT were noted (Table 3). Previous studies also demonstrated a higher population differentiation in *IL4 *between Europeans and East Asians [51]. These findings suggest that different selective factors might be exerting locus-specific effects in populations with different geographic origin. Most of the genes under local adaptation were in the 'immune response' and 'apoptosis' pathways (Table 3). How these biological processes may promote differential susceptibility to CVD among Africans and non-Africans need further study.

Given that CVD susceptibility varies among populations, genes that are responsible for such variations should also differ among populations. Hence, regardless of whether drift or selection is responsible, the approach of looking among a set of candidate genes for those with highest *F*_*ST *_values should help identify candidate genes to explain differences in CVD susceptibility. The present study identifies genes in etiological pathways of CVD with a high *F*_*ST *_among populations, and should be considered as a means of generating new hypotheses to test (i.e., reprioritize candidate genes rather than identify new ones). Putative functional SNPs with a high *F*_*ST *_should be investigated further for confirmation of functional effects and should be included in CVD association studies among populations. In addition, the differential patterns of *F*_*ST *_among biological processes and functional pathways may provide insight into the mechanisms contributing to varying CVD susceptibility among different populations.

Our study has several limitations. First, since population differentiation detects local adaptation in geographically separate populations within the last ~75,000 years [52], the present study cannot identify genes subject to natural selection before this time scale. Phylogenetic analyses [52] detect evolutionary changes preceding this time period whereas nucleotide diversity and LD-based tests [22,53] may be helpful in further investigating genomic regions with significantly high *F*_*ST *_values. Second, the fluctuation of allele frequencies due to a relatively small sample size could affect the robustness of our inferences. Thus, estimation of allele frequencies in a larger sample would be needed to confirm our results. Even though common SNPs (MAF > 0.1) that conformed to Hardy-Weinberg equilibrium (HWE) were included, we cannot completely address the issues of ascertainment bias of SNPs [43]. However, SNPs with MAF < 0.1 and not in HWE could have biological relevance since natural selection, not genotyping error also could lead to deviation from HWE. Third, we did not adjust for recombination rate in our analyses. We found a negative correlation between the weighted-average *F*_*ST *_and recombination rate (based on the recombination map of Kong et al. [54]), although the correlation was not statistically significant (analyses not shown). The recombination rate in genes with a high *F*_*ST *_listed in Figure 6 (except GRB2 and PRIK1) was below the average recombination rate (1cM/MB) across the human genome. Finally, a complete catalogue of etiologic pathways implicated in CVD is yet to be established.

## Conclusion

In summary, the present study of genes in etiologic pathways for CVD revealed greater population differentiation in putative functional SNPs in these genes, as well as significant variation in *F*_*ST *_based on different biological processes relevant to CVD. The biological processes of 'apoptosis' and 'lipoprotein metabolism' showed an excess of genes with high *F*_*ST*_. In addition, the pattern varied in different pairwise population comparisons. SNP loci (especially putatively functional SNPs) and genes with a significantly higher population differentiation should be considered high priority for investigating genetic factors influencing differences in CVD risk among populations.

## Methods

### Genes in the etiologic pathways for cardiovascular disease (CVD)

Based on a search of the literature in PUBMED [55], we identified 37 functional pathways implicated in CVD (a summary of the functional pathways, the number of genes in each pathway and corresponding references is presented in Table S1, see additional file 1). We explored 416 genes from these pathways using the Panther classification system [56,57]. These genes were classified into the following biological processes relevant to CVD: 1) apoptosis; 2) blood circulation & gas exchange; 3) blood clotting; 4) homeostasis; 5) immunity and defense; and 6) lipid fatty acid & steroid metabolism. Examples of candidate genes in various pathways were: caspase and TNF/TNF receptor gene family in the 'apoptosis' process; endothelin and nitric oxide synthase 3 genes in the 'blood circulation & gas exchange' process; those genes involved in the intrinsic and extrinsic coagulation pathway in the 'blood clotting' process; the gene family of insulin receptor substrate in the 'homeostasis' process; those genes participating in the inflammation response, such as the interleukin gene family, in the 'immunity and defense' process; and the arachidonate-lipoxygenase, and phospholipase gene family in the 'lipid fatty acid and steroid metabolism' process.

### Genotype data

Using the National Center for Biotechnology Information (NCBI) reference sequence [58], we aligned the sequence of messenger RNA of each gene with the human chromosome sequence (NCBI build 35). Based on the alignment, the genotype data for single nucleotide polymorphisms (SNPs) in each gene were obtained from HapMap database (Phase II) [24,33]. The HapMap data includes 90 individuals (30 trios) from the Yoruba in Ibadan, Nigeria (YRI), 90 individuals (30 trios) in Utah residents with ancestry from Northern and Western Europe (CEU), 45 unrelated Han Chinese in Beijing, China (CHB) and 45 unrelated Japanese in Tokyo, Japan (JPT). For each gene, map information for SNPs was obtained from NCBI and the genome annotation database at University of California, Santa Cruz (UCSC) [27]. The reference mRNA sequence was annotated as 5' untranslated regions (5' UTR), coding (synonymous and non-synonymous), intronic, and 3' untranslated regions (3' UTR).

### Calculation of *F*_*ST*_

The estimate of population differentiation, *F*_*ST*_, measures relatedness of pairs of alleles within a population relative to the total populations [16,35]. We calculated an unbiased small-sample estimator of *F*_*ST *_as described by Weir [16,59]. If there are *n*_*i *_alleles sampled from the *i*^*th *^of *r *populations, the sample frequency of the SNP allele *u *in the *i*^*th *^subpopulation is p˜iu
 MathType@MTEF@5@5@+=feaafiart1ev1aaatCvAUfKttLearuWrP9MDH5MBPbIqV92AaeXatLxBI9gBaebbnrfifHhDYfgasaacH8akY=wiFfYdH8Gipec8Eeeu0xXdbba9frFj0=OqFfea0dXdd9vqai=hGuQ8kuc9pgc9s8qqaq=dirpe0xb9q8qiLsFr0=vr0=vr0dc8meaabaqaciaacaGaaeqabaqabeGadaaakeaacuWGWbaCgaacamaaBaaaleaacqWGPbqAcqWG1bqDaeqaaaaa@311E@, and a weighted average of *p*_*u *_across population is p¯u=1∑ini∑i=1rnip˜iu
 MathType@MTEF@5@5@+=feaafiart1ev1aaatCvAUfKttLearuWrP9MDH5MBPbIqV92AaeXatLxBI9gBaebbnrfifHhDYfgasaacH8akY=wiFfYdH8Gipec8Eeeu0xXdbba9frFj0=OqFfea0dXdd9vqai=hGuQ8kuc9pgc9s8qqaq=dirpe0xb9q8qiLsFr0=vr0=vr0dc8meaabaqaciaacaGaaeqabaqabeGadaaakeaacuWGWbaCgaqeamaaBaaaleaacqWG1bqDaeqaaOGaeyypa0ZaaSaaaeaacqaIXaqmaeaadaaeqbqaaiabd6gaUnaaBaaaleaacqWGPbqAaeqaaaqaaiabdMgaPbqab0GaeyyeIuoaaaGcdaaeWbqaaiabd6gaUnaaBaaaleaacqWGPbqAaeqaaOGafmiCaaNbaGaadaWgaaWcbaGaemyAaKMaemyDauhabeaaaeaacqWGPbqAcqGH9aqpcqaIXaqmaeaacqWGYbGCa0GaeyyeIuoaaaa@46A2@. Two mean squares were defined as, MSGu=1∑i=1r(ni−1)∑i=1rnip˜iu(1−p˜iu)
 MathType@MTEF@5@5@+=feaafiart1ev1aaatCvAUfKttLearuWrP9MDH5MBPbIqV92AaeXatLxBI9gBaebbnrfifHhDYfgasaacH8akY=wiFfYdH8Gipec8Eeeu0xXdbba9frFj0=OqFfea0dXdd9vqai=hGuQ8kuc9pgc9s8qqaq=dirpe0xb9q8qiLsFr0=vr0=vr0dc8meaabaqaciaacaGaaeqabaqabeGadaaakeaacqWGnbqtcqWGtbWucqWGhbWrdaWgaaWcbaGaemyDauhabeaakiabg2da9maalaaabaGaeGymaedabaWaaabCaeaacqGGOaakcqWGUbGBdaWgaaWcbaGaemyAaKgabeaakiabgkHiTiabigdaXiabcMcaPaWcbaGaemyAaKMaeyypa0JaeGymaedabaGaemOCaihaniabggHiLdaaaOWaaabCaeaacqWGUbGBdaWgaaWcbaGaemyAaKgabeaakiqbdchaWzaaiaWaaSbaaSqaaiabdMgaPjabdwha1bqabaGccqGGOaakcqaIXaqmcqGHsislcuWGWbaCgaacamaaBaaaleaacqWGPbqAcqWG1bqDaeqaaOGaeiykaKcaleaacqWGPbqAcqGH9aqpcqaIXaqmaeaacqWGYbGCa0GaeyyeIuoaaaa@57D0@, and MSPu=1r−1∑i=1rni(p˜iu−p¯u)2
 MathType@MTEF@5@5@+=feaafiart1ev1aaatCvAUfKttLearuWrP9MDH5MBPbIqV92AaeXatLxBI9gBaebbnrfifHhDYfgasaacH8akY=wiFfYdH8Gipec8Eeeu0xXdbba9frFj0=OqFfea0dXdd9vqai=hGuQ8kuc9pgc9s8qqaq=dirpe0xb9q8qiLsFr0=vr0=vr0dc8meaabaqaciaacaGaaeqabaqabeGadaaakeaacqWGnbqtcqWGtbWucqWGqbaudaWgaaWcbaGaemyDauhabeaakiabg2da9maalaaabaGaeGymaedabaGaemOCaiNaeyOeI0IaeGymaedaamaaqahabaGaemOBa42aaSbaaSqaaiabdMgaPbqabaGccqGGOaakcuWGWbaCgaacamaaBaaaleaacqWGPbqAcqWG1bqDaeqaaOGaeyOeI0IafmiCaaNbaebadaWgaaWcbaGaemyDauhabeaakiabcMcaPmaaCaaaleqabaGaeGOmaidaaaqaaiabdMgaPjabg2da9iabigdaXaqaaiabdkhaYbqdcqGHris5aaaa@4C6F@, where *MSG*_*u *_and *MSP*_*u *_denote the observed mean square errors for loci with populations and between populations, respectively. The moment estimator of *F*_*ST *_was defined as, FST=MSPu−MSGuMSPu+(nc−1)MSGu
 MathType@MTEF@5@5@+=feaafiart1ev1aaatCvAUfKttLearuWrP9MDH5MBPbIqV92AaeXatLxBI9gBaebbnrfifHhDYfgasaacH8akY=wiFfYdH8Gipec8Eeeu0xXdbba9frFj0=OqFfea0dXdd9vqai=hGuQ8kuc9pgc9s8qqaq=dirpe0xb9q8qiLsFr0=vr0=vr0dc8meaabaqaciaacaGaaeqabaqabeGadaaakeaacqWGgbGrdaWgaaWcbaGaem4uamLaemivaqfabeaakiabg2da9maalaaabaGaemyta0Kaem4uamLaemiuaa1aaSbaaSqaaiabdwha1bqabaGccqGHsislcqWGnbqtcqWGtbWucqWGhbWrdaWgaaWcbaGaemyDauhabeaaaOqaaiabd2eanjabdofatjabdcfaqnaaBaaaleaacqWG1bqDaeqaaOGaey4kaSIaeiikaGIaemOBa42aaSbaaSqaaiabdogaJbqabaGccqGHsislcqaIXaqmcqGGPaqkcqWGnbqtcqWGtbWucqWGhbWrdaWgaaWcbaGaemyDauhabeaaaaaaaa@4E17@, where, *n*_*c *_is the average sample size across samples that also incorporates and corrects for the variance in sample size over subpopulations, nc=1r−1(∑i=1rni−∑i=1rni2∑i=1rni)
 MathType@MTEF@5@5@+=feaafiart1ev1aaatCvAUfKttLearuWrP9MDH5MBPbIqV92AaeXatLxBI9gBaebbnrfifHhDYfgasaacH8akY=wiFfYdH8Gipec8Eeeu0xXdbba9frFj0=OqFfea0dXdd9vqai=hGuQ8kuc9pgc9s8qqaq=dirpe0xb9q8qiLsFr0=vr0=vr0dc8meaabaqaciaacaGaaeqabaqabeGadaaakeaacqWGUbGBdaWgaaWcbaGaem4yamgabeaakiabg2da9maalaaabaGaeGymaedabaGaemOCaiNaeyOeI0IaeGymaedaaiabcIcaOmaaqahabaGaemOBa42aaSbaaSqaaiabdMgaPbqabaaabaGaemyAaKMaeyypa0JaeGymaedabaGaemOCaihaniabggHiLdGccqGHsisldaWcaaqaamaaqadabaGaemOBa42aa0baaSqaaiabdMgaPbqaaiabikdaYaaaaeaacqWGPbqAcqGH9aqpcqaIXaqmaeaacqWGYbGCa0GaeyyeIuoaaOqaamaaqadabaGaemOBa42aaSbaaSqaaiabdMgaPbqabaaabaGaemyAaKMaeyypa0JaeGymaedabaGaemOCaihaniabggHiLdaaaOGaeiykaKcaaa@55C9@.

*F*_*ST *_was estimated for each SNP locus and a weighted-average *F*_*ST *_was estimated for each gene [35,60]. *F*_*ST *_can be negative when levels of differentiation are close to zero and/or sample sizes are small, indicating no population differentiation at these loci [35]. In our analysis, we assigned a value of zero to negative *F*_*ST *_values. The program for calculating *F*_*ST *_for each SNP locus was written in Perl and is available from the authors upon request. The weighted-average *F*_*ST *_value combining information over loci [35] was calculated using the 'Genepop' software (version 3.4) [61].

### Expected distribution of *F*_*ST *_under a calibrated demographic model

We used coalescent theory to obtain the expected distribution of *F*_*ST *_under a calibrated demographic model for Africans, Europeans, and Asians [32]. Using the program 'cosi' [62], we simulated a one megabase (MB) region 1,000 times under the 'best-fitting' population parameters for the three populations. The 'best-fitting' set of parameters yielded good agreement with all aspects (including allele frequency spectrum, fraction of alleles that are ancestral, linkage disequilibrium, and *F*_*ST*_) of the observed data in the human genome [32]. Pairwise *F*_*ST *_among populations was calculated for each simulated SNP.

#### Significance of *F*_*ST*_

Population demographic history, such as migration among sub-populations, can also influence *F*_*ST *_[63]. By comparing the *F*_*ST *_of an individual locus to the empirical distribution, it is possible to distinguish between genetic drift and natural selection without having to take population demographic history into account [64]. To assess the statistical significance of *F*_*ST *_values for SNPs, we selected 289 intergenic regions across the autosomal genome and 17 intergenic regions across chromosome X to obtain a neutral distribution of *F*_*ST*_, based on the annotation tables of human chromosomes from UCSC database. These regions were separated by at least one MB from the closest exon and did not include centromeric regions. Each region spanned an average of 1.52 MB and in total composed 466.10 MB. Following the method of Izagirre *et al*. [47], we defined 'neutral' SNPs as follows: 1) separated by at least 25 kb from each other, 2) genotyped in all three populations, and 3) MAF ≥ 0.10 in at least one of the three populations. In all, 14,792 SNPs for autosomal chromosomes and 372 SNPs for the X chromosome satisfied these criteria. We used the observed 'neutral' distribution to assess the significance level of the *F*_*ST *_for each SNP (*P *value, one-sided) in the autosomal and X chromosome regions separately. We focused on the significantly higher values of *F*_*ST *_for local adaptation, although significantly lower *F*_*ST *_might result from balancing selection.

All statistical analyses were performed using *R*. Analysis of variance (ANOVA) was performed to compare linear regressions of *F*_*ST *_against logarithm of MAF [ln(MAF)] with and without the terms for the SNP category and a ln(MAF) × category interaction.

## Authors' contributions

IJK and KD designed the project, analyzed the data, and wrote the paper. Both authors read and approved the final manuscript.

## Supplementary Material

Additional file 1 Patterns of population differentiation of candidate genes for cardiovascular disease. Table S1 describes the summary of functional pathways implicated in atherosclerosis; Table S2 shows mean *F*_*ST *_values for SNPs of different categories, conditioned on MAF; and Table S3 is the summary of function for genes with a significant weighted-average *F*_*ST*_. Click here for file
