# Supplementary material for: Patterns of population differentiation of candidate genes for cardiovascular disease
Source: BMC Genet. 2007 Jul 12;8:48. doi: 10.1186/1471-2156-8-48 (PMC1937006; doi:10.1186/1471-2156-8-48)
Supplement: Additional file 1 — Patterns of population differentiation of candidate genes for cardiovascular disease. Table S1 describes the summary of functional pathways implicated in atherosclerosis; Table S2 shows mean FST values for SNPs of different categories, conditioned on MAF; and Table S3 is the summary of function for genes with a significant weighted-average FST. [file 1471-2156-8-48-S1.doc]

**Supplementary materials**

**Patterns of population differentiation of candidate genes for cardiovascular disease**

Iftikhar J. Kullo^1*^, Keyue Ding^1^

^1^ Division of Cardiovascular Diseases, Mayo Clinic, Rochester MN, USA

^*^ Corresponding author

Email addresses:

IJK: kullo.iftikhar@mayo.edu

KD: ding.keyue@mayo.edu

Table S1: Summary of functional pathways implicated in atherosclerosis

| **Pathways implicated in atherosclerosis** | **Number of genes** | **Reference** |
| --- | --- | --- |
| Alpha adrenergic receptor signaling pathway | 27 | [1] |
| Angiogenesis | 81 | [2] [3] |
| Apoptosis signaling pathway | 105 | [4] |
| B cell activation | 70 | [5] |
| Blood coagulation | 42 | [6] [7] |
| Cadherin signaling pathway | 8 | [8] |
| Carnitine metabolism | 1 | [9] |
| Cholesterol biosynthesis | 10 | [10] |
| EGF receptor signaling pathway | 66 | [11] |
| Endothelin signaling pathway | 50 | [12] |
| FAS signaling pathway | 25 | [13] |
| FGF signaling pathway | 46 | [14] |
| Heterotrimeric G-protein signaling pathway-Gi alpha and Gs alpha mediated pathway | 3 | [15] |
| Heterotrimeric G-protein signaling pathway-Gq alpha and Go alpha mediated pathway | 30 | [16] |
| Hypoxia response via HIF activation | 5 | [17] |
| Inflammation mediated by chemokine and cytokine signaling pathway | 145 | [18] |
| Insulin/IGF pathway-mitogen activated protein kinase kinase/MAP kinase cascade | 15 | [19] |
| Insulin/IGF pathway-protein kinase B signaling cascade | 16 | [20] |
| Integrin signalling pathway | 36 | [21] |
| Interferon-gamma signaling pathway | 18 | [22] |
| Interleukin signaling pathway | 50 | [23] |
| JAK/STAT signaling pathway | 15 | [24] |
| Metabotropic glutamate receptor group I pathway | 27 | [25] |
| Muscarinic acetylcholine receptor 1 and 3 signaling pathway | 34 | [26] |
| Muscarinic acetylcholine receptor 2 and 4 signaling pathway | 7 | [26] |
| Nicotinic acetylcholine receptor signaling pathway | 7 | [27] |
| Oxidative stress response | 28 | [28] |
| p53 pathway | 39 | [8] |
| PDGF signaling pathway | 63 | [29] |
| PI3 kinase pathway | 27 | [20] |
| Plasminogen activating cascade | 15 | [30] |
| Ras Pathway | 24 | [31] |
| T cell activation | 77 | [32] |
| TGF-beta signaling pathway | 11 | [33] |
| Toll receptor signaling pathway | 44 | [34] |
| Ubiquitin proteasome pathway | 1 | [35] |
| VEGF signaling pathway | 57 | [36] |

Table S2: Mean *F_ST_* values for SNPs of different categories, conditioned on MAF

| MAF | YRI vs. CEU | | | | | YRI vs. CHB + JPT | | | | | CEU vs. CHB + JPT | | | | |
| --- | --- | --- | --- | --- | --- | --- | --- | --- | --- | --- | --- | --- | --- | --- | --- |
|  | 5FR | 3FR | Synon | non-synon | intron | 5FR | 3FR | synon | non-synon | intron | 5FR | 3FR | synon | non-synon | intron |
| [0, 0.05) | 0.034 | 0.017 | 0.041 | 0.034 | 0.031 | 0.040 | 0.042 | 0.030 | 0.040 | 0.031 | 0.020 | 0.024 | 0.032 | 0.036 | 0.024 |
| [0.05, 0.10) | 0.074 | 0.076 | 0.072 | 0.086 | 0.077 | 0.101 | 0.107 | 0.100 | 0.090 | 0.089 | 0.086 | 0.079 | 0.065 | 0.088 | 0.076 |
| [0.10, 0.15) | 0.107 | 0.119 | 0.094 | 0.118 | 0.099 | 0.141 | 0.101 | 0.120 | 0.132 | 0.114 | 0.093 | 0.109 | 0.098 | 0.107 | 0.092 |
| [0.15, 0.20) | 0.124 | 0.102 | 0.127 | 0.157 | 0.126 | 0.150 | 0.143 | 0.140 | 0.146 | 0.143 | 0.076 | 0.100 | 0.097 | 0.149 | 0.098 |
| [0.20, 0.25) | 0.133 | 0.138 | 0.156 | 0.120 | 0.139 | 0.147 | 0.151 | 0.131 | 0.162 | 0.165 | 0.044 | 0.101 | 0.102 | 0.070 | 0.107 |
| [0.25, 0.30) | 0.174 | 0.133 | 0.124 | 0.205 | 0.147 | 0.210 | 0.170 | 0.163 | 0.221 | 0.175 | 0.122 | 0.143 | 0.070 | 0.111 | 0.115 |
| [0.30, 0.35) | 0.226 | 0.258 | 0.107 | 0.196 | 0.168 | 0.177 | 0.108 | 0.207 | 0.240 | 0.194 | 0.107 | 0.091 | 0.108 | 0.213 | 0.116 |
| [0.35, 0.40) | 0.182 | 0.149 | 0.244 | 0.178 | 0.200 | 0.182 | 0.154 | 0.124 | 0.303 | 0.201 | 0.097 | 0.147 | 0.095 | 0.150 | 0.119 |
| [0.40, 0.45) | 0.179 | 0.168 | 0.229 | 0.322 | 0.206 | 0.173 | 0.377 | 0.272 | 0.049 | 0.239 | 0.131 | 0.109 | 0.142 | 0.251 | 0.115 |
| [0.45, 0.50] | 0.174 | 0.221 | 0.194 | 0.504 | 0.204 | 0.187 | 0.187 | 0.204 | 0.316 | 0.252 | 0.141 | 0.039 | 0.078 | 0.177 | 0.099 |

Note. − 5FR, 5´ flanking regions; 3FR, 3´ flanking regions; synon, synonymous; non-synon, non-synonymous; MAF, minor allele frequency

Table S3: Functional properties of genes with a significant weighted-average *F_ST_*

| **Gene** | **OMIM** | **Gene function / implicated in CVD** | **Reference** |
| --- | --- | --- | --- |
| *GRB2* | 108355 | Essential for cardiac hypertrophy and fibrosis in response to pressure overload different signaling pathways downstream of GRB2 regulate fibrosis, fetal gene induction, and cardiomyocyte growth. | [37] |
| *IKBKB* | 603258 | *IKBKB*/*NF-κB* transcription pathway is a key regulator of *IL6*, *IL8*, and *TNFα* release from adipose tissue, and has a role in *TNFα*-induced *ICAM1* expression and induces cell-cell adhesion and aortic smooth muscle cell proliferation | [38-40] |
| *IL4* | 147780 | Activates the Janus kinase/signal transduction and transcription (JAK/STAT) signaling cascade | [41] |
| *IL6* | 147620 | Modulates downstream inflammatory and angiogenic targets, i.e., stimulates the production of large amounts of acute-phase reactants (CRP, serum amyloid A, and fibrinogen) | [42] |
| *ARHGEF1* | 601855 | Regulates cell proliferation | [43] |
| *RIPK1* | 603453 | Important not only in later phases of the immune response, when TNF is active, but also at the beginning, when an antiviral immune response is engaged via TLR3 interaction with double-stranded RNA | [44] |
| *BCL2L1* | 600039 | Deregulated expression of *BCL2L1* may contribute to the erythropoietin-independent survival of erythroid-lineage cells in polycythemia vera | [45] |
| *PMVK* | 607622 | In rat liver, *Pmvk* is regulated by dietary sterol levels and this regulation is coordinated with HMG-CoA reductase. *PMVK* possessed kinase activity with conversion of the monophosphate to the diphosphate. | [46, 47] |
| *F2* | 176930 | Mutations (32 molecular defects) in *F2* are involved in the pathogenesis of thrombophilia | [48] |

**References**

1. Baumgart D, Haude M, Gorge G, Liu F, Ge J, Grosse-Eggebrecht C, Erbel R, Heusch G: **Augmented alpha-adrenergic constriction of atherosclerotic human coronary arteries**. *Circulation* 1999, **99**:2090-2097.

2. Khurana R, Simons M, Martin JF, Zachary IC: **Role of angiogenesis in cardiovascular disease: a critical appraisal**. *Circulation* 2005, **112**:1813-1824.

3. Weber C: **Platelets and chemokines in atherosclerosis: partners in crime**. *Circ Res* 2005, **96**:612-616.

4. Brandes RP, Fleming I, Busse R: **Endothelial aging**. *Cardiovasc Res* 2005, **66**:286-294.

5. Binder CJ, Shaw PX, Chang MK, Boullier A, Hartvigsen K, Horkko S, Miller YI, Woelkers DA, Corr M, Witztum JL: **The role of natural antibodies in atherogenesis**. *J Lipid Res* 2005, **46**:1353-1363.

6. Mackman N: **Role of tissue factor in hemostasis, thrombosis, and vascular development**. *Arterioscler Thromb Vasc Biol* 2004, **24**:1015-1022.

7. Khrenov AV, Ananyeva NM, Griffin JH, Saenko EL: **Coagulation pathways in atherothrombosis**. *Trends Cardiovasc Med* 2002, **12**:317-324.

8. Ross JS, Stagliano NE, DonoVan MJ, Breitbart RE, Ginsburg GS: **Atherosclerosis and cancer: common molecular pathways of disease development and progression**. *Ann NY Acad Sci* 2001, **947**:271-293.

9. Hiatt WR: **Carnitine and peripheral arterial disease**. *Ann N Y Acad Sci* 2004, **1033**:92-98.

10. Waterham HR: **Inherited disorders of cholesterol biosynthesis**. *Clin Genet* 2002, **61**:393-403.

11. Kawakami A, Yoshida M: **Remnant lipoproteins and atherogenesis**. *J Atheroscler Thromb* 2005, **12**:73-76.

12. Barton M, Traupe T, Haudenschild CC: **Endothelin, hypercholesterolemia and atherosclerosis**. *Coron Artery Dis* 2003, **14**:477-490.

13. Geng YJ, Libby P: **Progression of atheroma: a struggle between death and procreation**. *Arterioscler Thromb Vasc Biol* 2002, **22**:1370-1380.

14. Eyries M, Collins T, Khachigian LM: **Modulation of growth factor gene expression in vascular cells by oxidative stress**. *Endothelium* 2004, **11**:133-139.

15. Siffert W: **G protein polymorphisms in hypertension, atherosclerosis, and diabetes**. *Annu Rev Med* 2005, **56**:17-28.

16. McLaughlin JN, Mazzoni MR, Cleator JH, Earls L, Perdigoto AL, Brooks JD, Muldowney JA, 3rd, Vaughan DE, Hamm HE: **Thrombin modulates the expression of a set of genes including thrombospondin-1 in human microvascular endothelial cells**. *J Biol Chem* 2005, **280**:22172-22180.

17. Kietzmann T, Roth U, Jungermann K: **Induction of the Plasminogen Activator Inhibitor-1 Gene Expression by Mild Hypoxia Via a Hypoxia Response Element Binding the Hypoxia-Inducible Factor-1 in Rat Hepatocytes**. *Blood* 1999, **94**:4177-4185.

18. Boyle JJ: **Macrophage activation in atherosclerosis: pathogenesis and pharmacology of plaque rupture**. *Curr Vasc Pharmacol* 2005, **3**:63-68.

19. Blanc A, Pandey NR, Srivastava AK: **Synchronous activation of ERK 1/2, p38mapk and PKB/Akt signaling by H2O2 in vascular smooth muscle cells: potential involvement in vascular disease (review)**. *Int J Mol Med* 2003, **11**:229-234.

20. Berk BC, Abe JI, Min W, Surapisitchat J, Yan C: **Endothelial atheroprotective and anti-inflammatory mechanisms**. *Ann N Y Acad Sci* 2001, **947**:93-109; discussion 109-111.

21. Delafontaine P, Song YH, Li Y: **Expression, regulation, and function of IGF-1, IGF-1R, and IGF-1 binding proteins in blood vessels**. *Arterioscler Thromb Vasc Biol* 2004, **24**:435-444.

22. Young JL, Libby P, Schonbeck U: **Cytokines in the pathogenesis of atherosclerosis**. *Thromb Haemost* 2002, **88**:554-567.

23. Von der Thusen JH, Kuiper J, Van Berkel TJC, Biessen EAL: **Interleukins in Atherosclerosis: Molecular Pathways and Therapeutic Potential**. *Pharmacol Rev* 2003, **55**:133-166.

24. Brasier AR, Recinos A, 3rd, Eledrisi MS: **Vascular inflammation and the renin-angiotensin system**. *Arterioscler Thromb Vasc Biol* 2002, **22**:1257-1266.

25. Dalton ML, Gadson PF, Jr., Wrenn RW, Rosenquist TH: **Homocysteine signal cascade: production of phospholipids, activation of protein kinase C, and the induction of c-fos and c-myb in smooth muscle cells**. *Faseb J* 1997, **11**:703-711.

26. De Meyer GR, Bult H, Van Hoydonck AE, Jordaens FH, Buyssens N, Herman AG: **Neointima formation impairs endothelial muscarinic receptors while enhancing prostacyclin-mediated responses in the rabbit carotid artery**. *Circ Res* 1991, **68**:1669-1680.

27. Kajiwara K, Yanagihara N, Tsutsui M, Yashiro A, Tasaki H, Nakashima Y, Izumi F: **Atherogenic lipoproteins inhibit catecholamine secretion in cultured bovine adrenal medullary cells**. *Naunyn Schmiedebergs Arch Pharmacol* 1998, **358**:308-314.

28. Sagi O, Wolfson M, Utko N, Muradian K, Fraifeld V: **p66ShcA and ageing: modulation by longevity-promoting agent aurintricarboxylic acid**. *Mech Ageing Dev* 2005, **126**:249-254.

29. Raines EW: **PDGF and cardiovascular disease**. *Cytokine Growth Factor Rev* 2004, **15**:237-254.

30. Hajjar KA, Deora A: **New concepts in fibrinolysis and angiogenesis**. *Curr Atheroscler Rep* 2000, **2**:417-421.

31. Antonio V, Brouillet A, Janvier B, Monne C, Bereziat G, Andreani M, Raymondjean M: **Transcriptional regulation of the rat type IIA phospholipase A2 gene by cAMP and interleukin-1beta in vascular smooth muscle cells: interplay of the CCAAT/enhancer binding protein (C/EBP), nuclear factor-kappaB and Ets transcription factors**. *Biochem J* 2002, **368**:415-424.

32. Muhlestein JB, Anderson JL: **Chronic infection and coronary artery disease**. *Cardiol Clin* 2003, **21**:333-362.

33. Agrotis A, Kalinina N, Bobik A: **Transforming growth factor-beta, cell signaling and cardiovascular disorders**. *Curr Vasc Pharmacol* 2005, **3**:55-61.

34. Tobias P, Curtiss LK: **Thematic review series: The Immune System and Atherogenesis. Paying the price for pathogen protection: toll receptors in atherogenesis**. *J Lipid Res* 2005, **46**:404-411.

35. Herrmann J, Ciechanover A, Lerman LO, Lerman A: **The ubiquitin-proteasome system in cardiovascular diseases-a hypothesis extended**. *Cardiovasc Res* 2004, **61**:11-21.

36. Kuwano M, Fukushi J, Okamoto M, Nishie A, Goto H, Ishibashi T, Ono M: **Angiogenesis factors**. *Intern Med* 2001, **40**:565-572.

37. Zhang S, Weinheimer C, Courtois M, Kovacs A, Zhang CE, Cheng AM, Wang Y, Muslin AJ: **The role of the Grb2-p38 MAPK signaling pathway in cardiac hypertrophy and fibrosis**. *J Clin Invest* 2003, **111**:833-841.

38. Lappas M, Yee K, Permezel M, Rice GE: **Sulfasalazine and BAY 11-7082 interfere with the nuclear factor-kappa B and I kappa B kinase pathway to regulate the release of proinflammatory cytokines from human adipose tissue and skeletal muscle in vitro**. *Endocrinology* 2005, **146**:1491-1497.

39. Huang WC, Chen JJ, Chen CC: **c-Src-dependent tyrosine phosphorylation of IKKbeta is involved in tumor necrosis factor-alpha-induced intercellular adhesion molecule-1 expression**. *J Biol Chem* 2003, **278**:9944-9952.

40. Chandrasekar B, Bysani S, Mummidi S: **CXCL16 signals via Gi, phosphatidylinositol 3-kinase, Akt, I kappa B kinase, and nuclear factor-kappa B and induces cell-cell adhesion and aortic smooth muscle cell proliferation**. *J Biol Chem* 2004, **279**:3188-3196.

41. Kelly-Welch AE, Hanson EM, Boothby MR, Keegan AD: **Interleukin-4 and interleukin-13 signaling connections maps**. *Science* 2003, **300**:1527-1528.

42. Chen Y, Pawlikowska L, Yao JS, Shen F, Zhai W, Achrol AS, Lawton MT, Kwok PY, Yang GY, Young WL: **Interleukin-6 involvement in brain arteriovenous malformations**. *Ann Neurol* 2006, **59**:72-80.

43. Hart MJ, Sharma S, elMasry N, Qiu RG, McCabe P, Polakis P, Bollag G: **Identification of a novel guanine nucleotide exchange factor for the Rho GTPase**. *J Biol Chem* 1996, **271**:25452-25458.

44. Meylan E, Burns K, Hofmann K, Blancheteau V, Martinon F, Kelliher M, Tschopp J: **RIP1 is an essential mediator of Toll-like receptor 3-induced NF-kappa B activation**. *Nat Immunol* 2004, **5**:503-507.

45. Silva M, Richard C, Benito A, Sanz C, Olalla I, Fernandez-Luna JL: **Expression of Bcl-x in erythroid precursors from patients with polycythemia vera**. *N Engl J Med* 1998, **338**:564-571.

46. Olivier LM, Chambliss KL, Gibson KM, Krisans SK: **Characterization of phosphomevalonate kinase: chromosomal localization, regulation, and subcellular targeting**. *J Lipid Res* 1999, **40**:672-679.

47. Chambliss KL, Slaughter CA, Schreiner R, Hoffmann GF, Gibson KM: **Molecular cloning of human phosphomevalonate kinase and identification of a consensus peroxisomal targeting sequence**. *J Biol Chem* 1996, **271**:17330-17334.

48. Akhavan S, De Cristofaro R, Peyvandi F, Lavoretano S, Landolfi R, Mannucci PM: **Molecular and functional characterization of a natural homozygous Arg67His mutation in the prothrombin gene of a patient with a severe procoagulant defect contrasting with a mild hemorrhagic phenotype**. *Blood* 2002, **100**:1347-1353.
